# Aspartate beta-hydroxylase domain containing 1 as a prognostic marker associated with immune infiltration in skin cutaneous melanoma

**DOI:** 10.1186/s12885-023-10625-8

**Published:** 2023-03-31

**Authors:** Shiquan Sun, Min Deng, Juan Wen, Xiaoyuan Chen, Jiaqi Xu, Yu Liu, Huanhuan Wan, Jin Wang, Leping Yan, Yong He, Yunsheng Xu

**Affiliations:** 1 Department of Dermatovenereology, The Seventh Affiliated Hospital, Sun Yat-sen University, Shenzhen, 518107 China; 2 Department of Liver Surgery, Sun Yat-sen University Cancer Center, Guangzhou, 510060 China; 3 State Key Laboratory of Oncology in South China, Collaborative Innovation Center for Cancer Medicine, Guangzhou, 510060 China; 4 School of Medicine, Southeast University, Nanjing, 211189 China; 5 Hepatobiliary Center, Key Laboratory of Liver Transplantation, NHC Key Laboratory of Living Donor Liver Transplantation, The First Affiliated Hospital of Nanjing Medical University, Chinese Academy of Medical Sciences, Nanjing, 210029 China; 6 Department of Hematology, Nanjing Drum Tower Hospital, The Affiliated Hospital of Nanjing University Medical School, Nanjing, 210008 China; 7 Guangdong Provincial Key Laboratory of Digestive Cancer Research, The Seventh Affiliated Hospital, Sun Yat-sen University, Shenzhen, 518107 China

**Keywords:** ASPHD1, Skin cutaneous melanoma, CTLA4, Tumor immune microenvironment, Biomarkers

## Abstract

**Background:**

Skin cutaneous melanoma (SKCM) is an extremely malignant tumor and accounts for the majority of skin cancer deaths. Aspartate beta-hydroxylase domain containing 1 (ASPHD1) may participate in cancer progression through controlling α-ketoglutarate-dependent dioxygenases. However, its role in skin cutaneous melanoma (SKCM) has not been well studied.

**Methods:**

The gene expression data of ASPDH1 and differentially expressed genes (DEGs) from TCGA and GTEx were evaluated, and verified via the GEO database. Then, we performed GO/KEGG, GSEA, PPI network analysis to analyze the functional implications of the DEGs related to ASPHD1. Then, the association between the ASPHD1 expression and clinical parameters was investigated by Cox regression analysis. Subsequently, the survival time of SKCM patients was evaluated by plotting Kaplan-Meier curves. Moreover, we investigated the correlation between the ASPHD1 expression and lymphocytic infiltration by using the data from TISIDB and TIMER 2.0. Next, we explored the association between ASPHD1 expression and drug sensitivity. Finally, we validate the expression differences by analyzing the results of qPCR, Western blot from human normal epidermal melanocytes and melanoma cells, and immunohistochemistry (IHC) from non-tumor skin as well as melanoma tissues.

**Results:**

The ASPHD1 expression level was significantly upregulated in several cancers, including SKCM especially SKCM-metastasis tissues, and patients with an increased ASPHD1 expression had longer overall survival time than low expression ones. The functional enrichment analysis of ASPHD1-related DEGs showed an association with cell development regulation and tumorigenic pathways. Furthermore, the increased ASPHD1 expression level was associated with the level of immunostimulors, immunoinhibitors, chemokines, and TILs, such as CD4^+^, CD8^+^ T cell, mast cell, Th2 cell, and dendritic cell. More interesting, we found that ASPHD1 expression was tightly associated with CTLA4 and CD276 which are immune checkpoint markers. Moreover, the upregulated expression of ASPHD1 exhibited higher IC50 values for 24 chemotherapy drugs, including doxorubicin, and masitinib. Finally, the differential expression of ASPHD1 in SKCM was validated by the results of qPCR, Western blot, and IHC.

**Conclusion:**

The expression of ASPHD1 in SKCM patients is closely related to patient survival. ASPHD1 may participate in the regulation of tumor immune microenvironment. Additionally, it may serve as a prognostic biomarker for SKCM and future in-depth studies are necessary to explore its value.

**Supplementary Information:**

The online version contains supplementary material available at 10.1186/s12885-023-10625-8.

## Background

Skin cutaneous melanoma (SKCM) is the most prevalent subtype of melanoma, and it is also the most lethal, accounting for most skin cancer deaths [1]. Although melanoma is a rare tumor compared to lung, breast, and gastrointestinal cancers, its incidence has increased rapidly in the past 40 years [2]. Even though there have been numerous new therapeutic agents introduced in recent years for unresectable melanoma and its overall survival rate has been significantly improved, the proportion of beneficiaries remains low and the treatment cost has increased significantly [2–5]. In most cases, the occurrence of skin cutaneous melanoma is related to high-level mutations caused by ultraviolet radiation [2]. Known genomic mutations in BRAF and NRAS are involved in melanoma formation [3, 6–8]. In addition, other pathways such as histone modification, methylation, and cell cycle change are also related to the occurrence of melanoma [9–11]. Although several biomarkers related to melanoma prognosis have been proposed [12], they are still controversial and have not been recognized. Therefore, it is urgently needed to discover new biomarkers for predicting melanoma recurrence, metastasis, and long-term survival.

ASPHD1 is a protein-coding gene whose related diseases include schizophrenia 3 and chromosome 16P11.2 Deletion Syndrome [13, 14]. The related gene annotation of Gene Ontology is dioxygenase activity. ASPHD1 is an important paralog of aspartate beta-hydroxylase (ASPH) whose gene coding protein is one of the type 2 transmembrane proteins, belongs to the family of α-ketoglutarate (αKG)-dependent dioxygenases [15]. The protein sequences and three dimensional structures from AlphaFold (predicted) for ASPH and ASPHD1 genes were showed in Figure S1. αKG-dependent dioxygenases can regulate tricarboxylic acid (TCA) cycle metabolites including αKG, 2-hydroxyglutarate, and fumarate, which are associated with chromatin demethylation [16]. ASPH participants in regulating calcium homeostasis and the malignant transformation of tumor cells [15]. The overexpression of ASPH in several human solid tumors, such as pancreatic ductal adenocarcinoma [17], breast cancer [18], colorectal cancer [19], cholangiocarcinoma [20], and hepatocarcinoma [21], has been observed. ASPH protein could catalyze posttranslational hydroxylation in the cbEGF-like domains of numerous proteins that are associated with cell motility and invasiveness [18]. Overexpressed ASPH proteins are transported from the endoplasmic reticulum to the plasma membrane, where their C-terminal region is exposed to the extracellular environment, and then it is possible to bind an antibody [21]. In addition, the antigenic epitopes of the ASPH protein can stimulate the cellular immune response by activating the Notch and SRC signaling pathways [15, 17, 18, 21]. Thus, ASPH is a potential candidate for molecular targeted therapy and immunotherapy. However, as a critical paralog of ASPH, the relationship between ASPHD1 and cancer is less studied, and its role in the occurrence and progression of neoplasm is not clear. Therefore, it is of significant importance to investigate the ASPHD1 expression level in different stages of SKCM and their correlations.

We investigated the association between ASPHD1 expression and the prognosis of SKCM patients by using several public databases in this study. Moreover, the relationship between ASPHD1 expression and lymphocytic infiltration in tumor immune microenvironments was also evaluated.

## Methods

### Patient data files

The original mRNA sequence profiles and clinical information of SKCM and non-tumor samples were obtained from TCGA (https://portal.gdc.cancer.gov/), GTEx (https://xena.ucsc.edu/), and GEO database (GSE114445, GSE15605, and GSE46517 datasets, https://www.ncbi.nlm.nih.gov/geo/). Then, sample data were subjected to normalization to minimize potential batch effects. In this study, gene expression profiles of 471 SKCM tissues and 558 nontumor tissues were analyzed using the TCGA and GTEx data. For validation, 30 normal skin and 93 primary melanoma gene expression profiles were obtained from three GEO datasets. Then, immunohistochemistry (IHC) of 4 normal human skin tissues and 2 melanoma tissues was obtained from the HPA database (http://www.proteinatlas.org/). 471 SKCM patient data from TCGA were used for statistical analysis including clinicopathological characteristics and overall survival. Table 1 provided details about the SKCM patients.

**Table 1:** Features of the SKCM patients in TCGA and GEO databases

Characteristics	Variable	TCGA cohort (N = 471)	GEO cohort (N = 93)
Age at diagnosis	≤ 58 years	238 (50.53)	36 (38.71)
	>58 years	225 (47.77)	57 (61.29)
	unknown	8 (1.70)	0 (0)
Sex	male	292 (62.00)	58 (62.37)
	Female	179 (38.00)	35 (37.63)
Vital status	Alive	249 (52.87)	NA
	death	221 (46.92)	NA
	unknown	1 (0.21)	
Pathological stage	0	6 (1.27)	0 (0)
	I-II	228 (48.41)	93 (100)
	III-IV	198 (42.04)	0 (0)
	unknown	39 (8.28)	0 (0)
T stage	Tis/ T0	30 (6.37)	0 (0)
	T1/ T2	120 (25.48)	57 (61.29)
	T3/ T4	243 (51.59)	36 (38.71)
	Unknown	78 (16.56)	0 (0)
N stage	N0	232 (49.26)	93 (100)
	N1	76 (16.14)	0 (0)
	N2	49 (10.40)	0 (0)
	N3	57 (12.10)	0 (0)
	Unknown	57 (12.10)	0 (0)
M stage	M0	416 (88.32)	93 (100)
	M1	25 (5.31)	0 (0)
	Unknown	30 (6.37)	0 (0)

SKCM, skin cutaneous melanoma. TCGA, The Cancer Genome Atlas. GEO, Gene Expression Omnibus. NA: not available. The median age at diagnosis was 58 years old in the TCGA database

### Identification of differentially expressed genes (DEGs)

471 SKCM patients were categorized into two groups based on median ASPHD1 expression levels. Then, DEGs were identified by using the “DESeq2” R package and ∣Log_2_ FC∣>1.5 as well as FDR < 0.05 were set. Then the heatmap map was performed by applying the “ggplot2” package.

### Functional enrichment analysis and gene set enrichment analysis (GSEA) of ASPHD1-related DEGs

The “limma” and “corrplot” R packages were used to identify the top 30 related DEGs. ASPHD1-related DEGs were analyzed using the “clusterProfiler” and “ggplot2” packages to investigate GO and KEGG functions. Subsequently, the ASPHD1-related pathways were assessed by using GSEA (version 4.1.0) in SKCM. An annotated gene set (C5. all. v7.1. symbols. gmt) was chosen as the reference. Gene expression enrichment analysis was carried out on ASPHD1 data sets with high or low expression. For each analysis, 1000 gene sets were set to identify significantly different pathways.

Three major parameters,∣standardized enrichment score∣>1, nominal p-value < 0.05 as well as error detection rate (FDR) q-value < 0.25 were regarded as statistically significant.

### Protein-protein interaction network (PPI) and gene co-expression network analysis of ASPHD1-associated DEGs

The genes related to ASPHD1 were input into the STRING database (http://string-db.org) (version 11.5) to evaluate the interaction between all the known proteins and explore potential mechanisms. As a minimum interaction fraction, medium confidence (0.400) was set to exclude irrelevant protein nodes. we used the Coexpedia database (http://www.coexpedia.org/) to analyze ASPHD1 co-expression genes in SKCM. Then, co-expressed genes were sorted and screened by neighbor’s local linear selection scores with a *p*-value < 0.05.

### Clinicopathological features associated with ASPHD1 expression in SKCM

We used Perl (Version 5.34.0) and R (Version 4.1.0) to sort, merge, and visualize the data derived from the TCGA and GTEx databases. Data from three GEO datasets were processed using similar methods. The SKCM patients were classified based on 50% ASPHD1 expression level. Then, an analysis of the relationship between ASPHD1 expression, survival status, and clinical features was performed. We applied Cox regression analyses to identify factors that affect overall survival (OS) in patients with SKCM by using R software.

### Association of ASPHD1 expression and immune-related factors in SKCM tumors

Integrated repository portals, TIMER 2.0 (http://timer.cistrome.org/) and TISIDB (http://cis.hku.hk/TISIDB/index.php) were used to evaluate the interactions between the tumor and immune system. They were used to investigate the ASPHD1 expression in different tumors and the relationship between the expression level of ASPHD1 and gene markers of TILs including CD4^+^/CD8^+^ T cell, T helper cell, dendritic cell (DC), B cell, and mast cell in this study. In addition, we studied the relationship of ASPHD1 with immunoinhibitors, immunostimulors, and chemokines in SKCM.

### Drug sensitivity prediction

IC50 of chemotherapy agent represents the half amount of an agent inhibiting some specific biological procedures. We used the “pRRophetic” R package to predict the IC50 of various chemotherapy drugs.

### Cell culture

Three human melanoma cells (A375, SK-MEL-28, and A875) as well as human normal epidermal melanocyte (HEMn) were provided by iCell Bioscience Co., Ltd (Shanghai, China). The absence of contamination of the three cell lines was confirmed by short tandem repeat analysis (Biowing applied biotechnology Co., Ltd, Shanghai, China). Routine examinations confirmed that the cells did not contain *mycoplasma*. DMEM, MEM, RPMI 1640, and DMEM F12 medium with adding 10% FBS and 1% penicillin-streptomycin were used to culture them under the conditions with 5% CO_2_ and 90% humidity at 37℃, respectively. All cell culture products were provided by Thermo Fisher Scientific Inc. (Waltham, MA, USA).

### Quantitative real-time PCR analysis

The processes of extracting the total RNA (Invitrogen, Thermo Fisher Scientific Inc., Waltham, MA, USA), the reverse transcription (Takara Bio Inc., Kusatsu, Japan), and real-time quantitative polymerase chain reaction analyses (Yeasen Biotechnology Co., Ltd., Shanghai, China) for mRNA of ASPHD1 and actin-beta were performed as previously described [22]. The 2^−ΔΔCt^ method was used to determine the relative expression levels of the target genes.

### Primers for qPCR were as follows

Human ASPHD1-F: 5′−CCAGAGTGGAATGTGGAAGGGAAAC − 3′.

Human ASPHD1-R: 5′−AGCAGTACCAGAGGAACAGGGAAG − 3′.

Human actin-beta-F: 5′−TCGTGCGTGACATTAAGGAGAAGC − 3′.

Human actin-beta-R: 5′− GGCGTACAGGTCTTTGCGGATG − 3′.

### Western blot

A lysis buffer was applied to extract the total protein of cells, then a BCA assay kit was used to evaluate the protein concentrations. All kits were provided by Beyotime Biotechnology Co., Ltd (Shanghai, China). Western blot was performed as previously described [22]. Anti-ASPHD1 (Abcam, cat#ab197301, Waltham, MA, USA) and anti-β-Tubulin (Abbkine Scientific Co., Ltd, cat#A01030, Wuhan, China) were used with the recommended concentrations. The blots were cut prior to hybridisation with antibodies.

### Statistical analysis

Mann–Whitney U test was used to compare the difference in ASPHD1 expression between SKCM and nontumor samples. The relationship between ASPHD1 expression and clinicopathological parameters was evaluated by chi-square test. The difference in survival time of SKCM patients was evaluated by plotting Kaplan-Meier curves. Univariate and multivariate analyses were performed by cox proportional hazard regression model. R software (version 4.1.0) was applied to conduct statistical analyses and *p* < 0.05 was considered significant in all trials.

## Results

### Patient characteristics and ASPHD1 expression in SKCM tissues

**Fig. 1:**
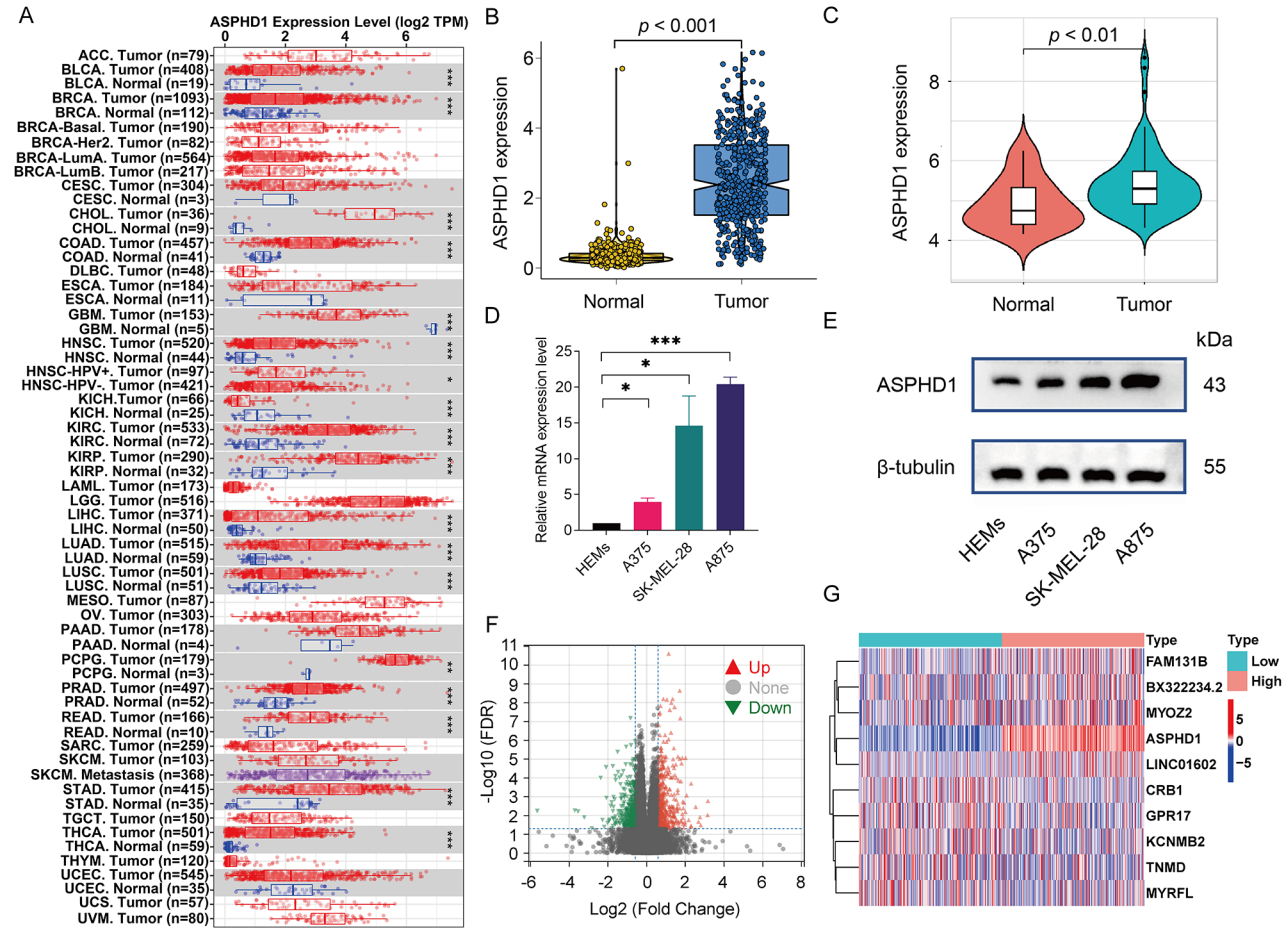
Differential mRNA expression profiles in skin cutaneous melanoma (SKCM) patients according to the ASPHD1 expression. **(A)** The expression of ASPHD1 in different tumors from the Tumor Immune Estimation Resource (TIMER 2.0). ASPHD1 expression was higher in SKCM tissues than in nontumor tissues based on TCGA and GTEx databases **(B)** as well as the GEO database **(C)**. The results of qPCR **(D)** and western blot **(E)** from human normal epidermal melanocyte (HEMn) and three human melanoma cell lines (A375, SK-MEL-28, and A875). 471 SKCM patients were divided into low and high expression groups according to the median ASPHD1 level, the differentially expressed genes (DEGs) were recognized and plotted by volcano **(F)** and heatmaps **(G)**. *p < 0.05; ***p < 0.001; ****p < 0.0001

According to the TIMER 2.0 database, the differential expression of ASPHD1 in various cancers was showing in Fig. 1A. The ASPHD1 expression levels were significantly upregulated in 15 kinds of cancers, including gastrointestinal cancer, hepatobiliary carcinoma, urological tumor, lung cancer, and breast invasive carcinoma, but decreased in glioblastoma multiforme compared with normal tissues.

We could also find that ASPHD1 expressed higher in SKCM, especially in SKCM metastasis tissues, but the compared result could be hardly obtained because there were not enough paired normal tissues with SKCM tissues in the TCGA database. To verify these findings in SKCM, the RNA-seq data of 471 SKCM samples were derived from TCGA and 558 nontumor samples including 1 from TCGA and 557 from GTEx were analyzed. The result showed that ASPHD1 expression in SKCM tissues was significantly elevated compared to tissues from non-tumors (Fig. 1B). A similar result was also verified by the GEO database (Fig. 1C). Meanwhile, mRNA and protein levels of ASPHD1 from three human melanoma cell lines were much higher than that of human normal epidermal melanocytes according to qRT-PCR (Fig. 1D) and western blot (Fig. 1E).

We divided 471 patients with SKCM into low and high groups based on 50% ASPHD1 expression. Then, we compared the mRNA levels between the two groups. We identified a total of 82 DEGs (Fig. 1F), including 49 upregulated genes and 33 downregulated genes. Then we plotted a heatmap displaying 10 representative DEGs (Fig. 1G).

### Functional enrichment analysis of ASPHD1-related DEGs

**Fig. 2:**
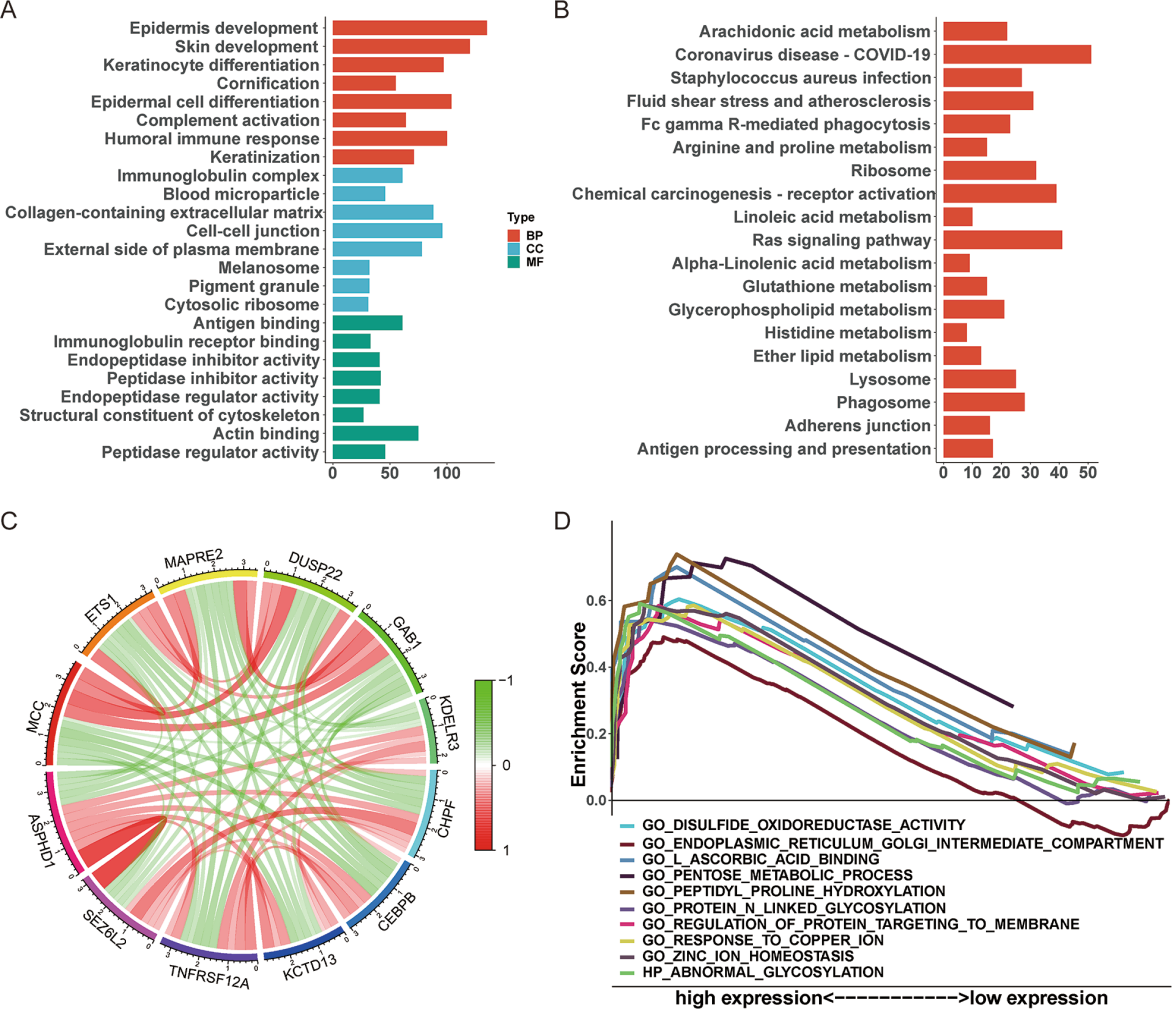
Functional enrichment analysis of ASPHD1-related DEGs in SKCM. **(A)** Annotations of GO enrichment analysis include biological process (BP), cellular components (CC), and molecular function (MF) categories. **(B)** KEGG pathway annotations, copyright permission of KEGG pathway maps was obtained from Kanehisa Laboratories. **(C)** Top 10 correlation genes of ASPHD1. Red means positive regulation and green means negative regulation. **(D)** Merged enrichment plots from the gene set enrichment analysis (GSEA).

We performed the GO (Fig. 2A) and KEGG (Fig. 2B) functional enrichment analysis to reveal the functional implication of ASPHD1-related DEGs. The relationship with the biological process (BP) included epidermal cell differentiation, skin development, and epidermis development; cellular components (CC) contained cell − cell junction, external side of plasma membrane and collagen-containing; molecular function (MF) involved peptidase regulator activity, antigen binding, and actin binding. KEGG included chemical carcinogenesis-receptor activation, Ras signaling pathway, and coronavirus disease [23–25]. Then, the 10 most correlated DEGs were plotted by the “limma” R package (Fig. 2C). The result of GSEA showed that the most enriched signaling pathways were peptidyl proline hydroxylation, disulfide oxidoreductase activity, regulation of protein targeting to membrane, L-ascorbic acid binding, pentose metabolic process, protein N-linked glycosylation, abnormal protein glycosylation, endoplasmic reticulum golgi intermediate compartment, zinc ion homestasis, response to copper ion. (Fig. 2D).

### Protein-protein interaction network (PPI) and gene co-expression network analysis

**Fig. 3:**
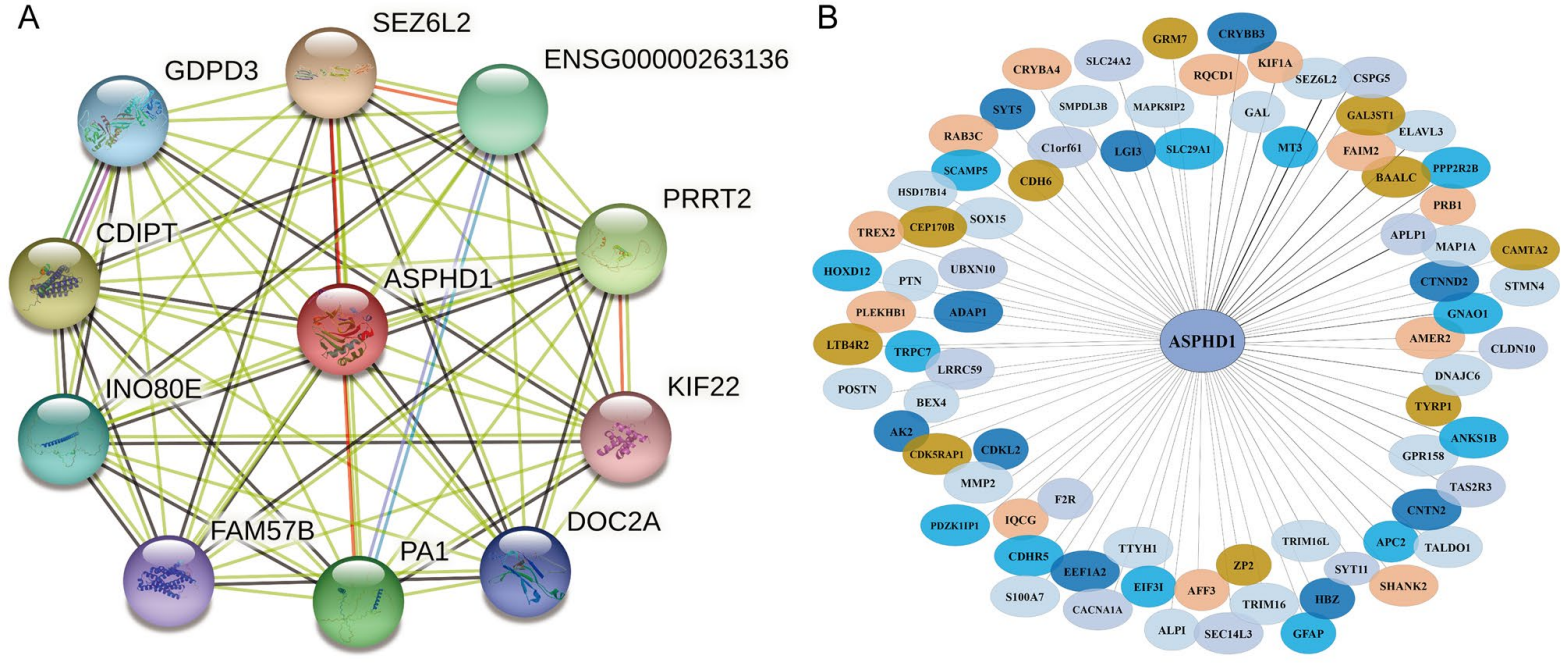
**(A)** The neighbor gene network was performed by protein-protein interaction network using STRING. **(B)** The gene co-expression networks of ASPHD1 in SKCM were conducted by the Coexpedia database

We used the STRING database to carry out PPI analysis to further investigate the mechanisms, and the results showed that 10 genes interacted with ASPHD1 including GDPD3, CDIPT, INO80E, FAM57B, PA1, DOC2A, KIF22, PRRT2, ENSG00000263136, and SEZ6L2 (Fig. 3A). Then, we identified the co-expression genes of ASPHD1 by the Coexpedia database, and it was found that 78 genes were associated with ASPHD1 (Fig. 3B).

### Correlation of ASPHD1 expression and clinicopathological characteristics

**Fig. 4:**
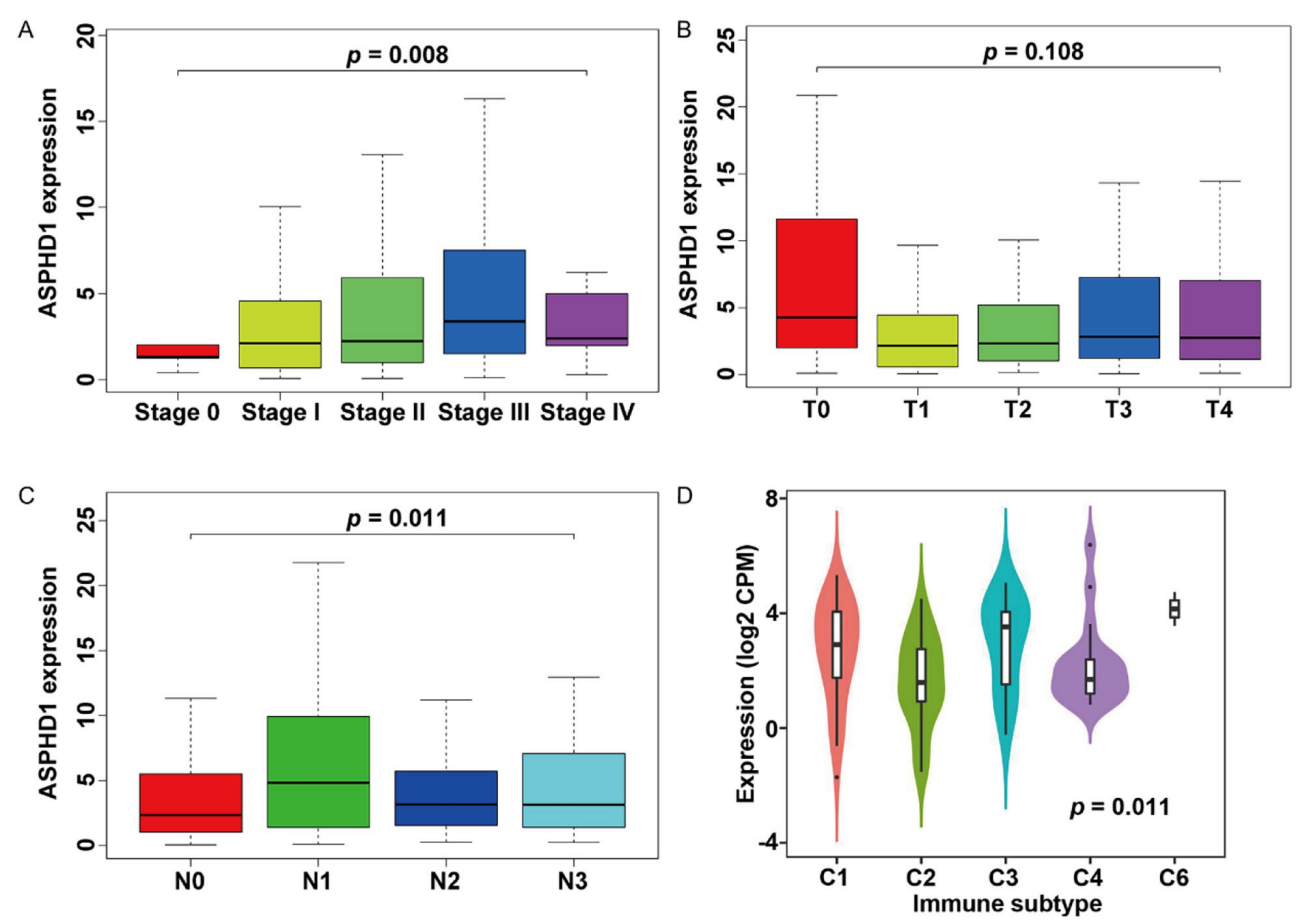
Correlation between ASPHD1 expression level and clinicopathological characteristics in SKCM patients. **(A)** Pathological stage. **(B)** Tumor stage (T). **(C)** Lymph node stage (N). **(D)** Immune subtype: C1 – wound healing, n = 41; C2 – IFN-gamma dominant, n = 27; C3 – inflammatory, n = 14; C4 – lymphocyte depleted, n = 19; C6 – TGF-b dominant, n = 2

The relationship between the expression of ASPHD1 and clinicopathological characteristics, including AJCC stage (stage0, I-IV), tumor stage (T0-T4), lymph node stage (N0-3), and immune subtypes (C1-6) was investigated. The results were shown in Fig. 4A-D. The results indicated that ASPHD1 expression was related to the pathological stage (III vs. 0, *p* = 0.047); T stage (II vs. 0, *p* = 0.033) as well as N stage (I vs. 0, *p* = 0.009) (Table 2).

**Table 2:** Logistic regression analysis of the relevance between ASPHD1 expression and clinicopathological parameters in SKCM

Clinicopathological parameters		Total (N)	OR (95%CI)	*p*-value
Age at diagnosis (years)	> 58 vs. ≤58	463	0.9741 (0.6753–1.4048)	0.8881
Sex	Male vs. Female	471	0.9071 (0.6241–1.3174)	0.6084
Pathological stage	I vs. 0	84	3.9130 (0.6253–75.8607)	0.2171
	II vs. 0	143	5.2 (0.8578–99.5641)	0.1316
	III vs. 0	180	8.7826 (1.4566-167.8178)	**0.0465**
	IV vs. 0	30	5.5 (0.7675-112.7308)	0.1410
T classification	I vs. 0	72	0.3580 (0.1153–1.0307)	0.0634
	II vs. 0	108	0.3381 (0.1183–0.8860)	**0.0326**
	III vs. 0	120	0.4574 (0.1624–1.1812)	0.1176
	IV vs. 0	183	0.4492 (0.1646–1.1160)	0.0963
Lymph nodes	I vs. 0	308	2.0536 (1.2084–3.5406)	**0.0085**
	II vs. 0	281	1.6071 (0.8620–3.032)	0.1373
	III vs. 0	289	1.3462 (0.7436–2.4452)	0.3257
Metastasis	M1 vs. M0	441	1.0048 (0.4369–2.3110)	0.9908

OR, odds ratio; CI, confidence interval. Bold values indicate *p* < 0.05; the median age at diagnosis was 58 years old. There were some missing data

### Association of ASPHD1 expression and overall survival time in SKCM patients

**Fig. 5:**
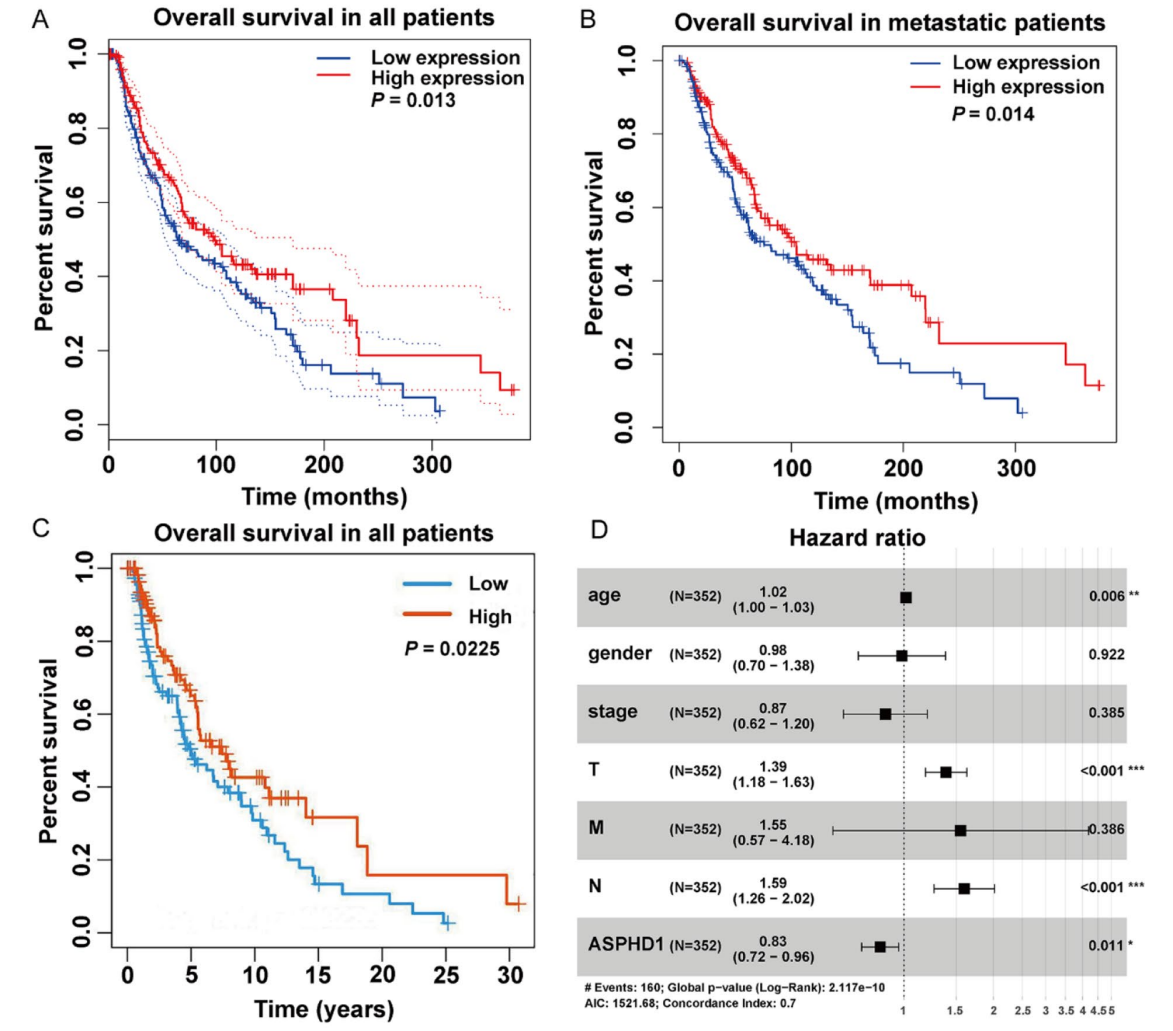
Kaplan-Meier curves comparing the low and high ASPHD1 expression in SKCM patients. Survival curves of overall survival (OS) in all SKCM patients **(A)** and metastatic patients **(B)** from TCGA. **(C)** Survival curves of OS in SKCM from TISIDB. **(D)** Forest plot for the multivariate Cox proportional hazard regression model. * *p* < 0.05, ** *p* < 0.01, *** *p* < 0.001

According to the median of the ASPHD1 expression level, high and low expression was grouped. The Kaplan-Meier curves revealed that the patients in the high group had better overall survival (OS) than the ones in the low group (Fig. 5A). The median OS in the high group was 42.2 months, while that in the low group was 37.1 months. The 5-year survival rates in the high and low groups were 66.1% and 54.6%, respectively. The result from the TISIDB database had a similar result (Fig. 5B). Moreover, the survival of metastatic patients with upregulated ASPHD1 had a better OS than the low ones (Fig. 5C).

Then, univariate analysis revealed that age, pathological stage, T stage, N stage, and ASPHD1 expression were potential survival predictors (Table 3). Multivariate analysis showed that age, T stage, N stage, and ASPHD1 expression were independent predictors of OS (Table 3; Fig. 5D).

**Table 3:** Univariate analysis and multivariate analysis of the clinicopathological parameters, the ASPHD1 expression, and overall survival among SKCM patients

Parameters	Univariate analysis			Multivariate analysis		
	HR	95%CI	*p*-value	HR	95%CI	*p*-value
Age	1.0207	1.0098–1.0316	**0.0002**	1.0151	1.0043–1.0261	**0.0062**
Sex	1.0539	0.7560–1.4693	0.7568	0.9832	0.7013–1.3784	0.9216
Pathological stage	1.4248	1.1920–1.7032	**0.0001**	0.8655	0.6248–1.1987	0.3847
T stage	1.3607	1.1851–1.5623	**< 0.0001**	1.3854	1.1792–1.6277	**< 0.0001**
N stage	1.4482	1.2394–1.6920	**< 0.0001**	1.5948	1.2609–2.0171	**< 0.0001**
M stage	1.7572	0.7177–4.3025	0.2172	1.5506	0.5748–4.1833	0.3863
ASPHD1	0.8677	0.7603–0.9903	**0.0353**	0.8307	0.7204–0.9580	**0.0107**

### Relationship of tumor-infiltrating lymphocytes and ASPHD1 in SKCM

**Fig. 6:**
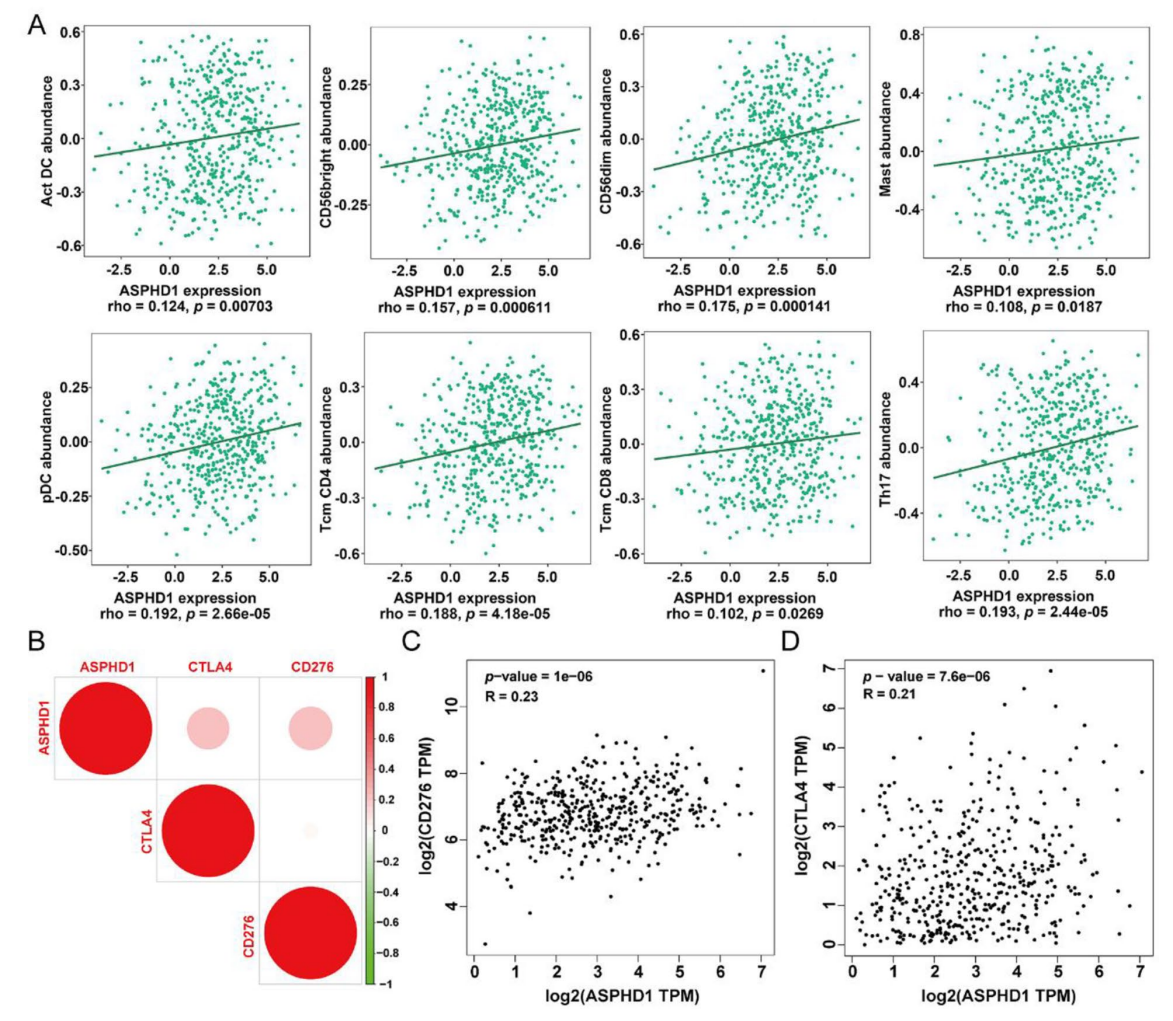
Correlation of ASPHD1 expression with immune infiltration and immune checkpoint in SKCM patients. **(A)** Correlation of the expression of ASPHD1 with infiltration levels of central memory CD4^+^ T cell, central memory CD8^+^ T cell, CD56 bright natural killer cell, CD56 dim natural killer cell, mast cell, activated dendritic cell, plasmacytoid dendritic cell and type 17 T helper cell. **(B)** ASPHD1 expression correlated with immune checkpoint CTLA4 and CD276. **(C)** Relation of the expression of ASPHD1 with CD276. **(D)** Correlation of the expression of ASPHD1 with CTLA4.

The TISIDB platform was applied to evaluate the association between the ASPHD1 expression level and TILs in SKCM. High-expression level of ASPHD1 had a positive relationship with central memory CD4^+^ T cell (*p* < 0.001), central memory CD8^+^ T cell (*p* = 0.026), CD56 bright natural killer cell (*p* < 0.001), CD56 dim natural killer cell (*p* < 0.001), mast cell (*p* = 0.018), activated dendritic cell (*p* = 0.007), plasmacytoid dendritic cell (*p* < 0.001) and type 17 T helper cell (*p* < 0.001) as shown in Fig. 6A. Moreover, increased ASPHD1 expression was positively correlated with immune checkpoint CTLA4 (*p* < 0.001) and CD276 (*p* < 0.001) in SKCM (Fig. 6B-D) according to the TIMER 2.0 database.

### Association of immunomodulators and ASPHD1 in SKCM

**Fig. 7:**
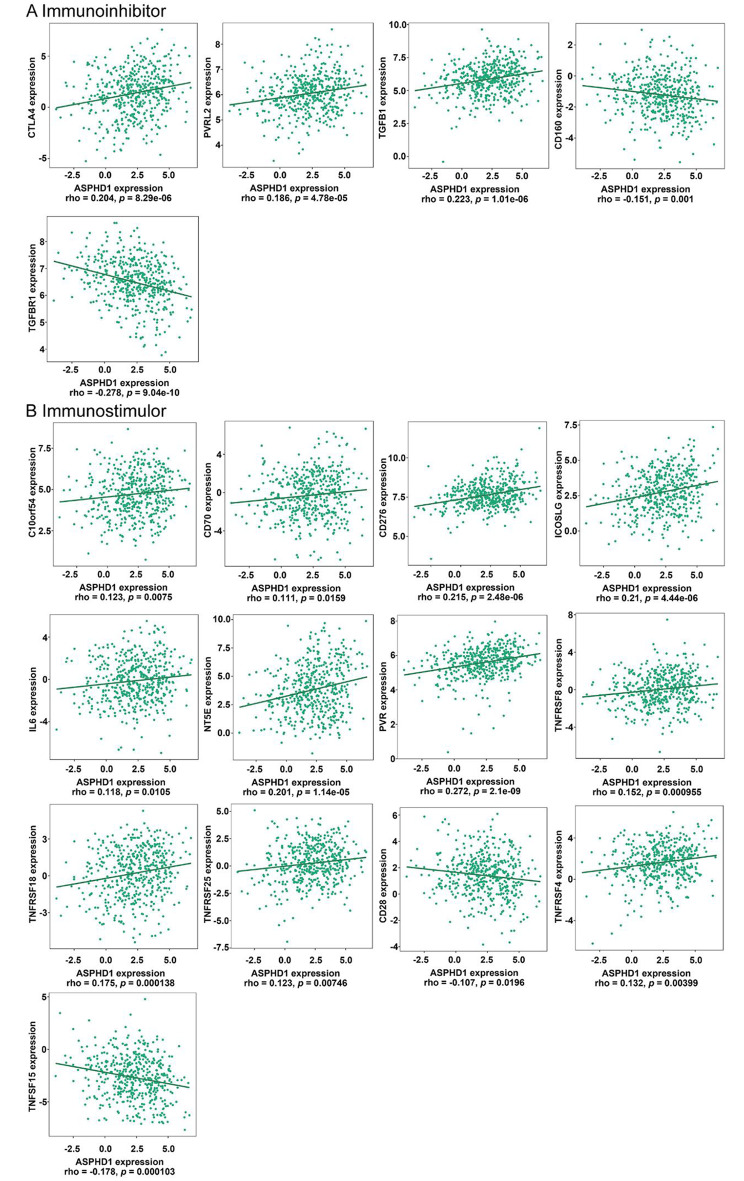
Association of ASPHD1 expression and immunomodulators in SKCM. **(A)** Correlation between ASPHD1 expression and immunoinhibitors in SKCM from TISIDB. **(B)** Correlation between ASPHD1 expression and immunostimulators in SKCM from TISIDB.

Immunoinhibitors and immunostimulors are clearly related to the function of the immune cell of the tumor immune microenvironment. The results in Fig. 7A showed that ASPHD1 was associated with immunoinhibitors, including PVRLL2 (*p* < 0.001), CTLA4 (*p* < 0.001), TGFB1(*p* < 0.001), TGFBR1 (*p* < 0.001) and CD160 (*p* = 0.001). The high-expressed ASPHD1 had a close association with immunostimulors, such as C10orf54 (*p* = 0.008), CD70 (*p* = 0.016), CD276 (*p* < 0.001), ICOSLG (*p* < 0.001), IL6 (*p* = 0.011), NTSE (*p* < 0.001), PVR (*p* < 0.001), TNFRSR8 (*p* = 0.001), TNFRSR18 (*p* < 0.001), TNFRSF25 (*p* = 0.007), CD28 (*p* = 0.020), TNFRSF4 (*p* = 0.004), TNFSF15 (*p* < 0.001) (Fig. 7B).

### The ASPHD1 expression is related to chemokines in SKCM

**Fig. 8:**
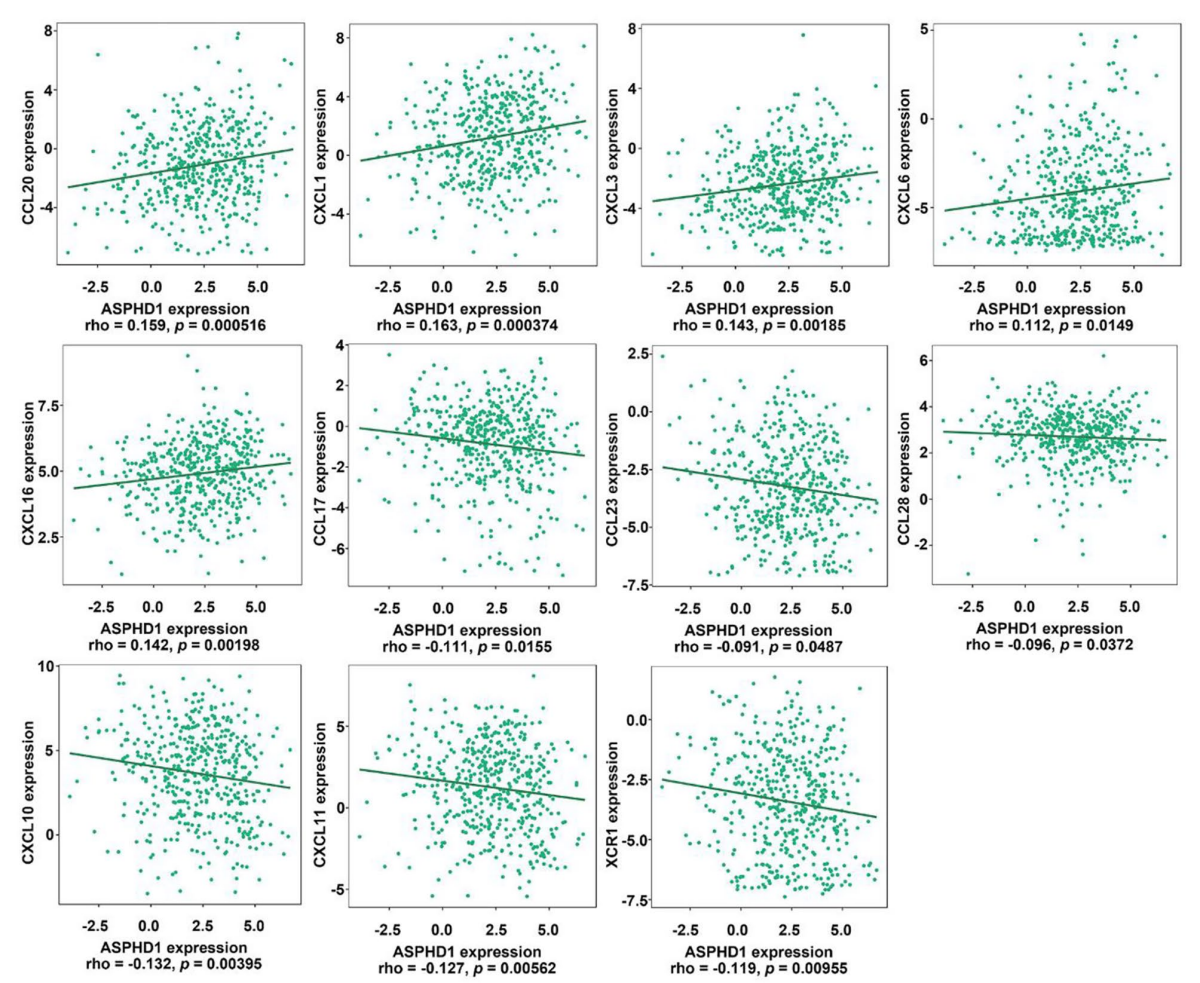
Correlation of ASPHD1 expression and chemokines in SKCM from TISIDB.

Chemokines play an important role in TILs infiltration and affect their function. This study uncovered the association between ASPHD1 expression and chemokines. As shown in Fig. 8, ASPHD1 was associated with CCL20 (*p* = 0.001), CXCL1 (*p* < 0.001), CXCL3 (*p* = 0.002), CXCL6 (*p* = 0.015) and CXCL16 (*p* = 0.002), CCL17 (*p* = 0.016), CCL23 (*p* = 0.048), CCL28 (*p* = 0.037), CXCL10 (*p* = 0.004), CXCL11 (*p* = 0.006), XCR1 (*p* = 0.010).

### Association of ASPHD1 expression and drug sensitivity in SKCM

**Fig. 9:**
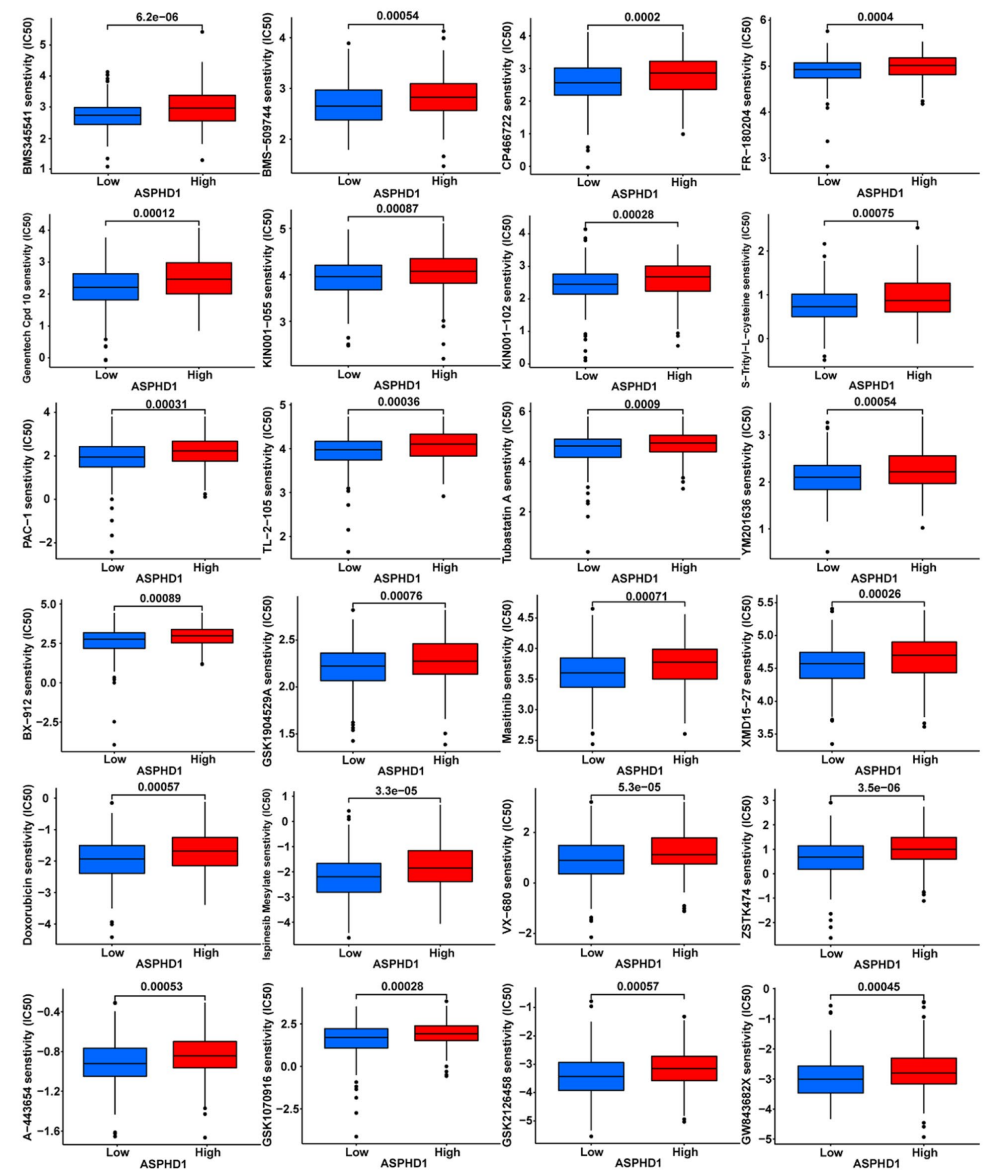
Drug sensitivity prediction in SKCM patients. Boxplots indicate the differences in estimated IC50 values of 24 representative drugs or inhibitors between the low and high ASPHD1 expression groups

The IC50 values of chemotherapy drugs were compared to investigate the difference in the drug resistance potential in low and high ASPHD1 expression groups. Figure 9 showed that 24 drugs or inhibitors, including doxorubicin and masitinib, were identified to be potential drugs for ASPHD1-downregulated SKCM patients.

### Validation of ASPHD1 expression

**Fig. 10:**
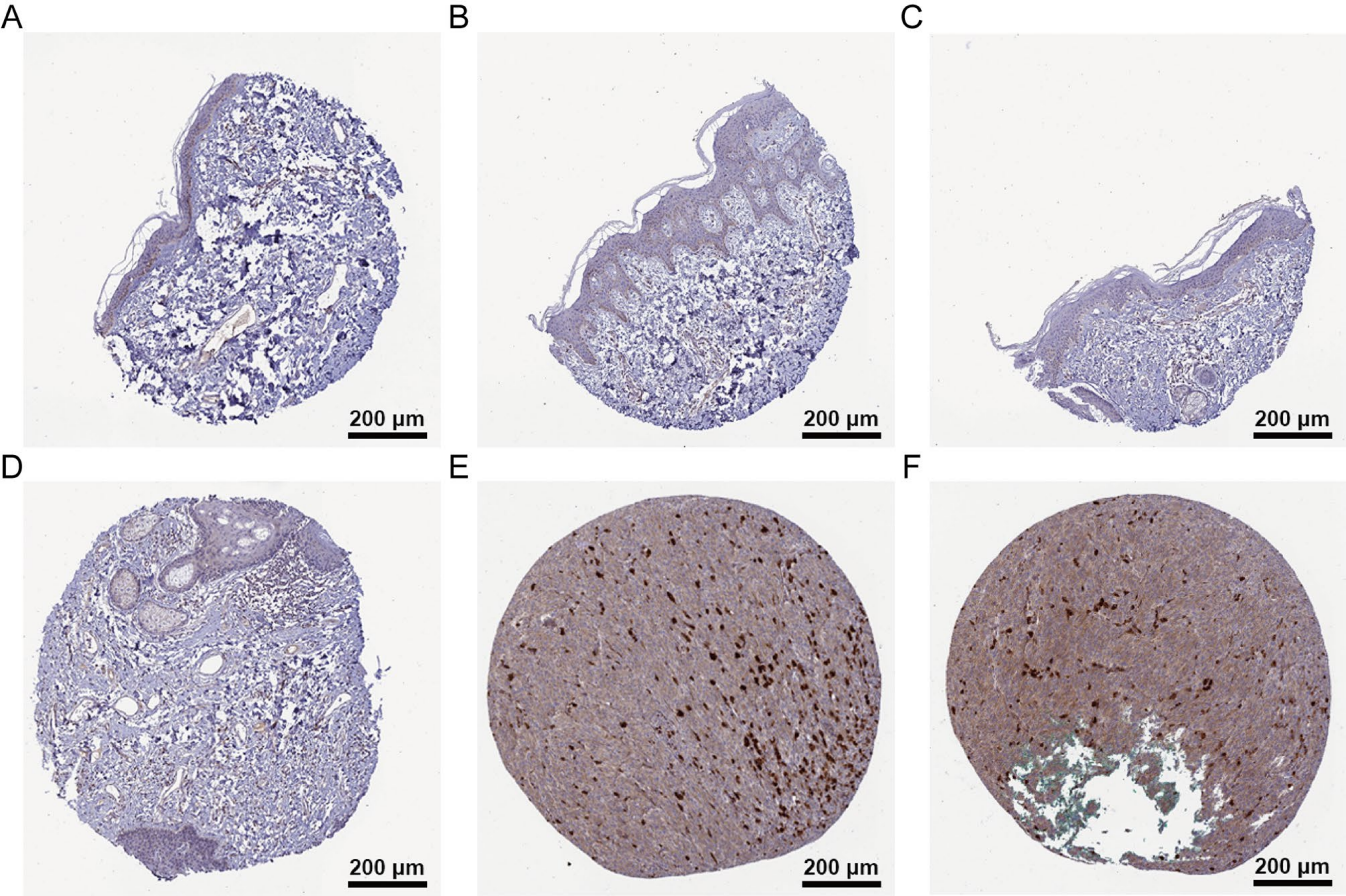
Representative Immunohistochemistry (IHC) staining images of ASPHD1 in normal skin and SKCM tissues. **(A-D)** ASPHD1 was not detected or low expression (< 25%) in normal skin tissues. **(E-F)** ASPHD1 was moderate expression (> 75%) in SKCM tissues

The IHC results indicated that ASPHD1 was not detected or low expressed in normal skin tissues and cells including Langerhans, fibroblasts, keratinocytes, melanocytes, and epidermal cells (Fig. 10A-D), but increased significantly the expression in melanoma (Fig. 10E-F).

## Discussion

An increasing number of studies have been published and have shown that ASPH is related to the malignant transformation and invasiveness of tumor cells [15, 17–21]. In addition, small-molecule inhibitors aimed at protecting against ASPH have been developed [15]. Therefore, ASPH is one of the most important biological targets to control tumor cells’ migration and invasion. However, the ASPHD1 expression level and its value in SKCM tissues are still unclear. In this work, we comprehensively analyzed the relationship between ASPHD1 expression and the clinical parameters, overall survival, and tumor immune microenvironment in SKCM. Analyzed results showed that ASPHD1 expression in SKCM tissues was upregulated, and patients with increased expression of ASPHD1 had a better prognosis, and the ASPHD1 expression level was an independent prognostic index along with age, T stage, and N stage on survival. Furthermore, the ASPHD1 expression was correlated with TILs, immunostimulors, immunoinhibitors, and chemokines as well as the sensitivity of several chemotherapy drugs. Therefore, the results of our study suggested that ASPHD1 might be a biomarker of prognosis that was related to the regulation of the immune microenvironment and a potential therapy target for SKCM.

Then, we investigated the biological functions of ASPHD1 in SKCM by functional enrichment analysis. ASPHD1 is closely related to αKG-dependent dioxygenases, which participates in maintaining stem cell properties to control cell fate by regulating tricarboxylic acid (TCA) cycle metabolites [26, 27]. The results from the functional annotation of ASPHD1-related DEGs showed that the ASPHD1 expression was related to epidermis development, skin development, and epidermal cell differentiation. Next, GSEA was conducted to identify the closely related signaling pathways of ASPHD1 in the development of SKCM. Proline hydroxylation is one of post-translational modifications and is related to 2-oxoglutarate (2OG) oxygenases which could catalyze the hydroxylation of proteins then regulate the basic cellular processes. Moreover, they are in-volved in many kinds of diseases, especially cancer [28]. N-linked glycosylation is a post-translational modification of proteins and is associated with ligand-receptor interactions [29]. Some studies have shown that programmed death-ligand 1 (PD-L1) in human tumors is glycosylated with heavy N-linked glycan moieties to maintain its protein stability and interaction with programmed cell death protein 1 (PD-1) [29]. Ascorbate could activate αKG-dependent dioxygenases to regulate cancer cell fate [30]. Zinc and copper are two kinds of metal elements that are essential for human physi-ology by regulating cell proliferation, catalysis, gene expression [31]. In addition, zinc is crucial for the immune system [32, 33] and copper is related to the synthesis of melanin pigment [34]. Their metabolic homeostasis is closely regulated in the body, so abnormal homeostasis has been reported in lots of cancers including melanoma [31–34]. Evidence showed that melanoma cells (A375) released fibroblast growth factor (FGF)-1 and FGF-2 and this process required copper ions by activation of phosphati-dylinositol 3-kinase (PI3K)/Akt pathway [35]. In conclusion, ASPHD1 is associated with different signaling pathways which involve in the occurrence and progression of SKCM.

Furthermore, we performed PPI and gene co-expression analyses to explore the related proteins and genes. The results showed that most of them are cancer-related. GDPD3 is necessary to maintain the stem cells of chronic myelogenous leukemia (CML). In addition, GDPD3 loss leads to the activation of AKT/mTORC1 signal and the progression of cell cycle [36]. In gastric cancer, CDIPT plays a crucial role in tumorigenesis and progression [37]. Some studies have suggested the functions of PA1 and INO80E in DNA damage repair and transcription regulation [38, 39]. KIF22, PRRT2, and SEZ6L2 are also related to several kinds of human solid tumors through different signaling pathways [40–42]. Therefore, we could hypothesize that ASPHD1 played a vital role in tumorigenesis and development.

Subsequently, we revealed that ASPHD1 was associated with the TILs infiltration in SKCM, and ASPHD1 participated in regulating TME. The results of this study showed that there was a strong correlation between ASPHD1 expression and the infiltration of CD4^+^ T cell, CD8^+^ T cell, natural killer cell, dendritic cell, mast cell, and Th17 cell. Moreover, increased ASPHD1 expression had a positive association with immune checkpoints CTLA4 and CD276 in SKCM, and upregulated ASPHD1 was associated with immunostimulors, immunoinhibitors, and chemokines. As a homolog of ASPHD1, ASPH contains both HLA I- and II- restriction epitopes, therefore it can induce ASPH antigen-specific CD4^+^ and CD8^+^ T cell responses, resulting in antitumor effects [16, 43]. To date, the relationship between ASPHD1 and cancers has been less studied, and its role in the occurrence and development of cancers is not clear. Therefore, it is of significant importance to explore the expression level and effect of ASPHD1 on prognosis in SKCM. When it was overexpressed in SKCM tissues along with the progression of SKCM, ASPHD1 protein could be transported to the cell surface and recognized by the immune system to stimulate antitumor immunity. This explains why the ASPHD1 expression level in SKCM tissues is much higher, and the prognosis of the high ASPHD1 expressed patients is better than that of low ASPHD1 expression ones.

Furthermore, the results of qPCR, western blot, and IHC validated that ASPHD1 expressed higher in SKCM cells and tissues compared to normal skin cells and tissues. Despite its consistency with our previous analysis, this study still has certain limitations. First, because partial clinical data are incomplete and perhaps related to the prognosis of SKCM, it is difficult to assess the relationship between ASPHD1 and these clinical characteristics of SKCM. Next, cases with incomplete clinicopathological data were filtered to obtain complete cases, which may lead to unsatisfactory results. For example, the relationship between the M stage and prognosis was inconsistent with the current clinical guidelines, probably due to the small number of patients with metastasis in the TCGA database. Finally, as far as we know, there have been a few studies focusing on ASPH and cancers, such as pancreatic ductal adenocarcinoma, hepatocellular carcinoma, and cholangiocarcinoma, but this is the first report on the relationship between ASPHD1 and melanoma. Hence, further research should be carried out on the role of ASPHD1 in cancer.

## Conclusion

The increased ASPHD1 expression is associated with better survival in SKCM, which is perhaps related to the higher level of TILs infiltration, immunostimulatory factors, and chemokines. Together, our study suggests ASPHD1 may be a potential biomarker for SKCM. Targeting ASPHD1 to regulate the progress of SKCM may be one of the potential directions, which could provide an essential theoretical basis for the precise treatment of SKCM. In the future, more biological experiments are needed to explore the function of ASPHD1, and it is necessary to carry out clinical trials to verify its value in the diagnosis and treatment of SKCM.

## Electronic supplementary material

Below is the link to the electronic supplementary material.

Supplementary Material 1

Supplementary Material 2

## Data Availability

Publicly available datasets were analyzed in this study, and these can be found in the Cancer Genome Atlas (https://portal.gdc.cancer.gov/), the Genotype-Tissue Expression (GTEx) (https://xena.ucsc.edu/), the Gene Expression Omnibus (GEO) database (GSE114445, GSE15605, and GSE46517 datasets, https://www.ncbi.nlm.nih.gov/geo/), and the Human Protein Atlas (http://www.proteinatlas.org/).

## Abbreviations

ASPHD1: Aspartate Beta-Hydroxylase Domain Containing 1
SKCM: Skin cutaneous melanoma
TCGA: The Cancer Genome Atlas
GTEx: Genotype-Tissue Expression
GEO: Gene Expression Omnibus
DEGs: Differentially expressed genes
GSEA: Gene set enrichment analysis
PPI: Protein-protein interaction
qPCR: Quantitative real-time polymerase chain reaction
IHC: Immunohistochemistry
OS: Overall survival
TILs: Tumor-infiltrating lymphocytes
HEMn: Human normal epidermal melanocytes
BRAF: V-raf murine sarcoma viral oncogene homolog B1
NRAS: Neuroblastoma Ras virus oncogene homolog
ASPH: Aspartate beta-hydroxylase
αKG: α-ketoglutarate
TCA: Tricarboxylic acid
DMEM: Dulbecco’s Modified Eagle’s Medium
IC50: Half maximal inhibitory concentration
PD-1: Programmed cell death protein 1
PD-L1: Programmed death-ligand 1
CML: Chronic myelogenous leukemia.
