# Supplementary material for: Aspartate beta-hydroxylase domain containing 1 as a prognostic marker associated with immune infiltration in skin cutaneous melanoma
Source: BMC Cancer. 2023 Mar 31;23:292. doi: 10.1186/s12885-023-10625-8 (PMC10063950; doi:10.1186/s12885-023-10625-8)
Supplement: Supplementary file 1 — Supplementary Material 1 [file 12885_2023_10625_MOESM1_ESM.docx]

**Aspartate beta-hydroxylase domain containing 1 as a prognostic marker associated with immune infiltration in skin cutaneous melanoma**

Shiquan Sun^1,†^, Min Deng^2,3,†^, Juan Wen^1,†^, Xiaoyuan Chen^4,5^, Jiaqi Xu^1^, Yu Liu^1^, Huanhuan Wan^1^, Jin Wang^6^, Leping Yan^7,*^, Yong He^1,*^, Yunsheng Xu^1,*^

**Supporting information**


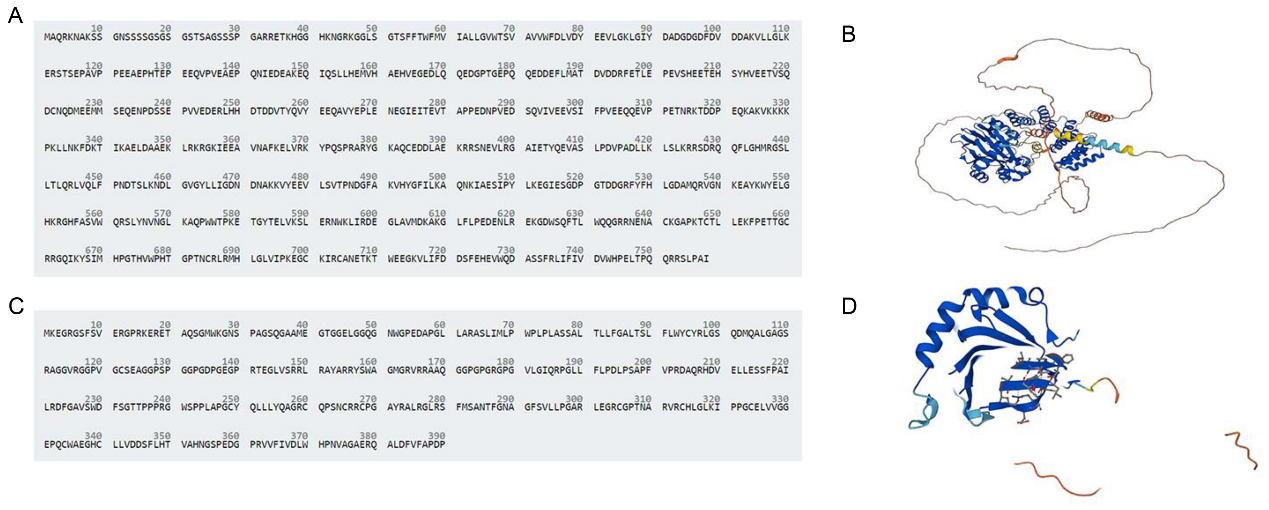


**Figure S1.** The protein sequences and three-dimensional structures from AlphaFold (predicted). The protein sequences for ASPH (isoform: Q12797-1) **(A)** and ASPHD1 **(C)**. The three-dimensional structures from AlphaFold (predicted) for ASPH (AF-Q12797-1) **(B)** and ASPHD1 (AF-Q5U4P2-1) **(D)**.


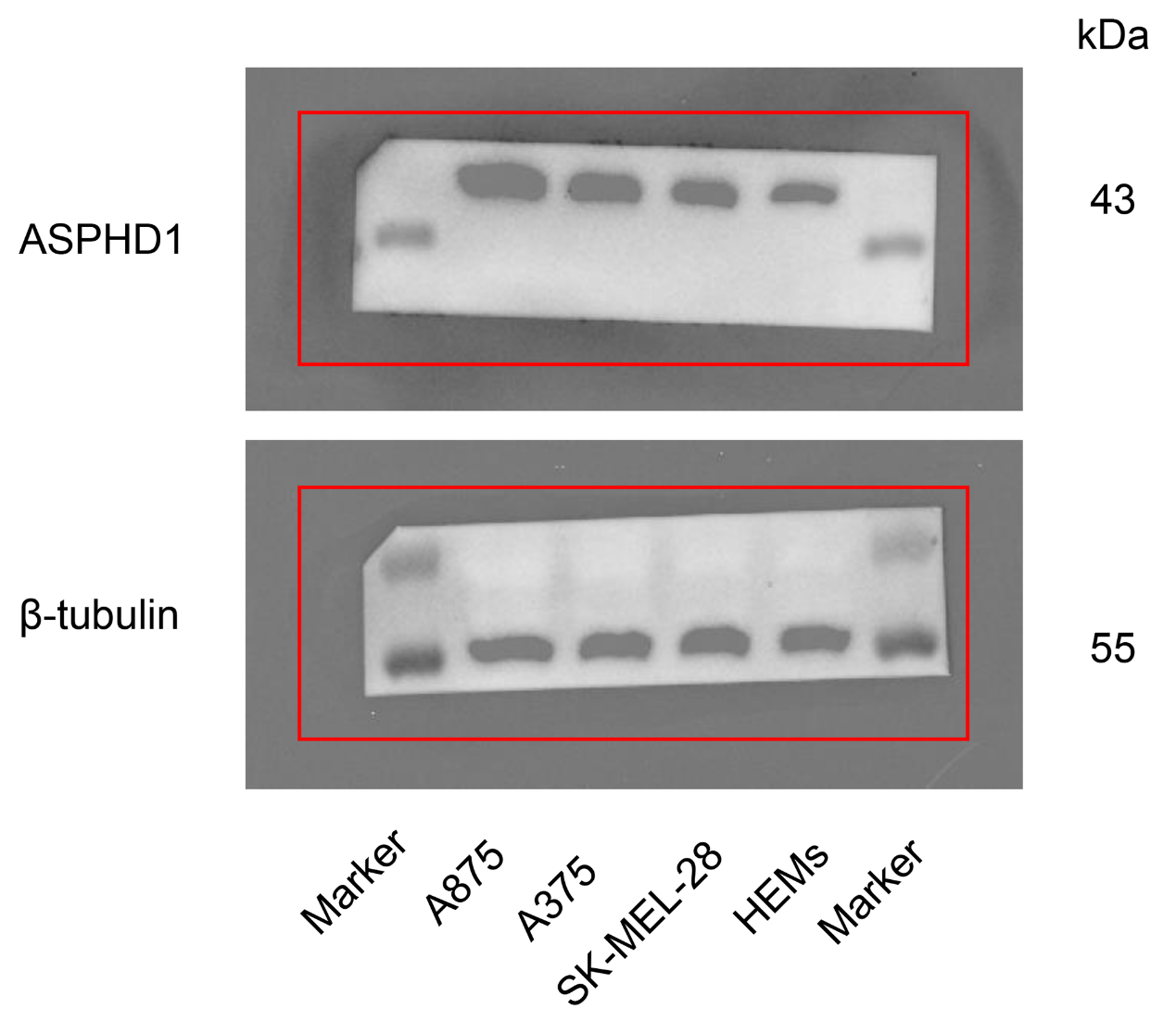


**Figure S2.** The original blots of ASPHD1 and β-tubulin which is consistent with Figure 1E in the manuscript. Because the blots were cut before hybridisation with antibodies, the original images of full-length blots were absent.


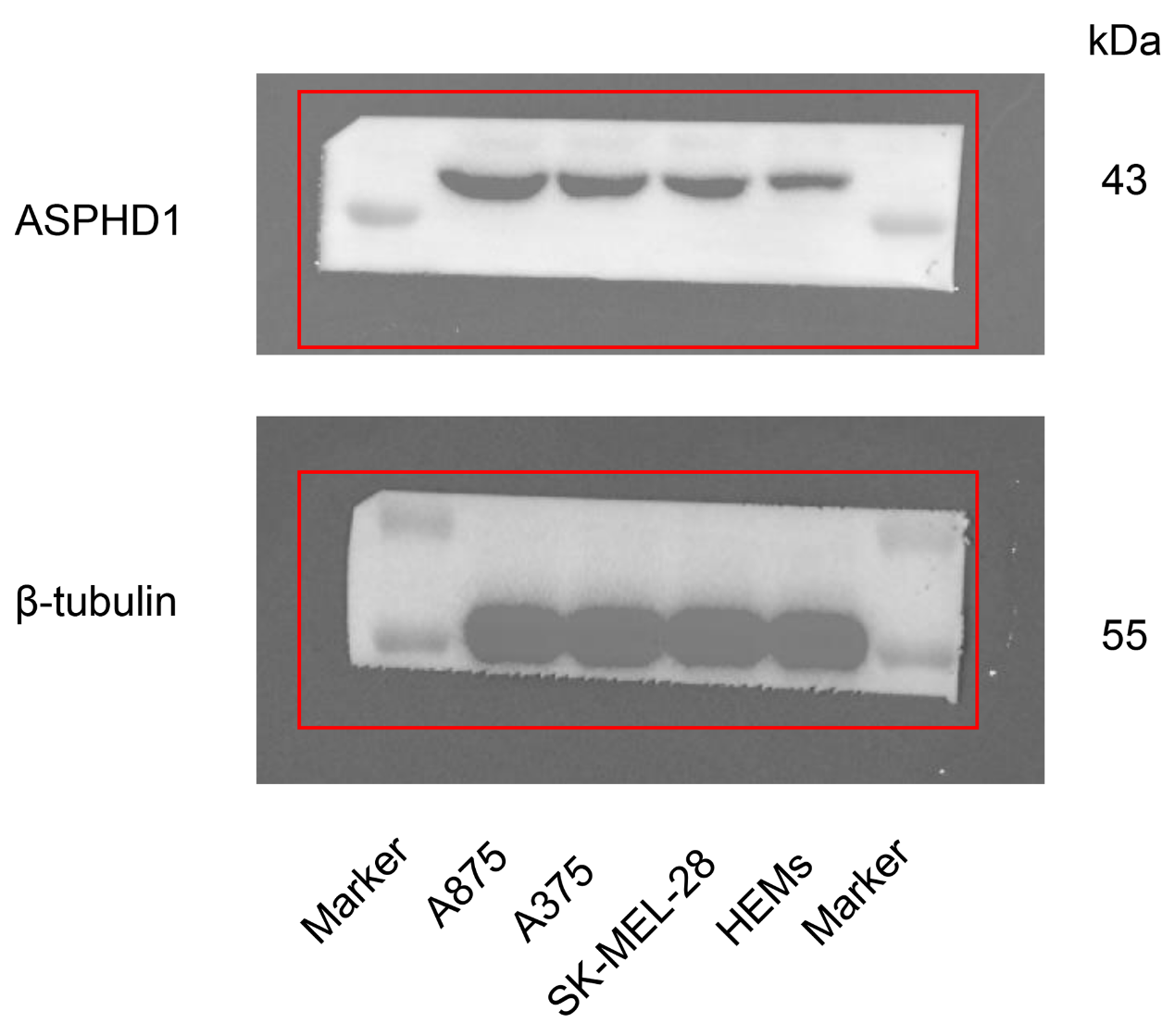


**Figure S3.** The original blots of ASPHD1 and β-tubulin for the second replicated experiment. Because the blots were cut before hybridisation with antibodies, the original images of full-length blots were absent.


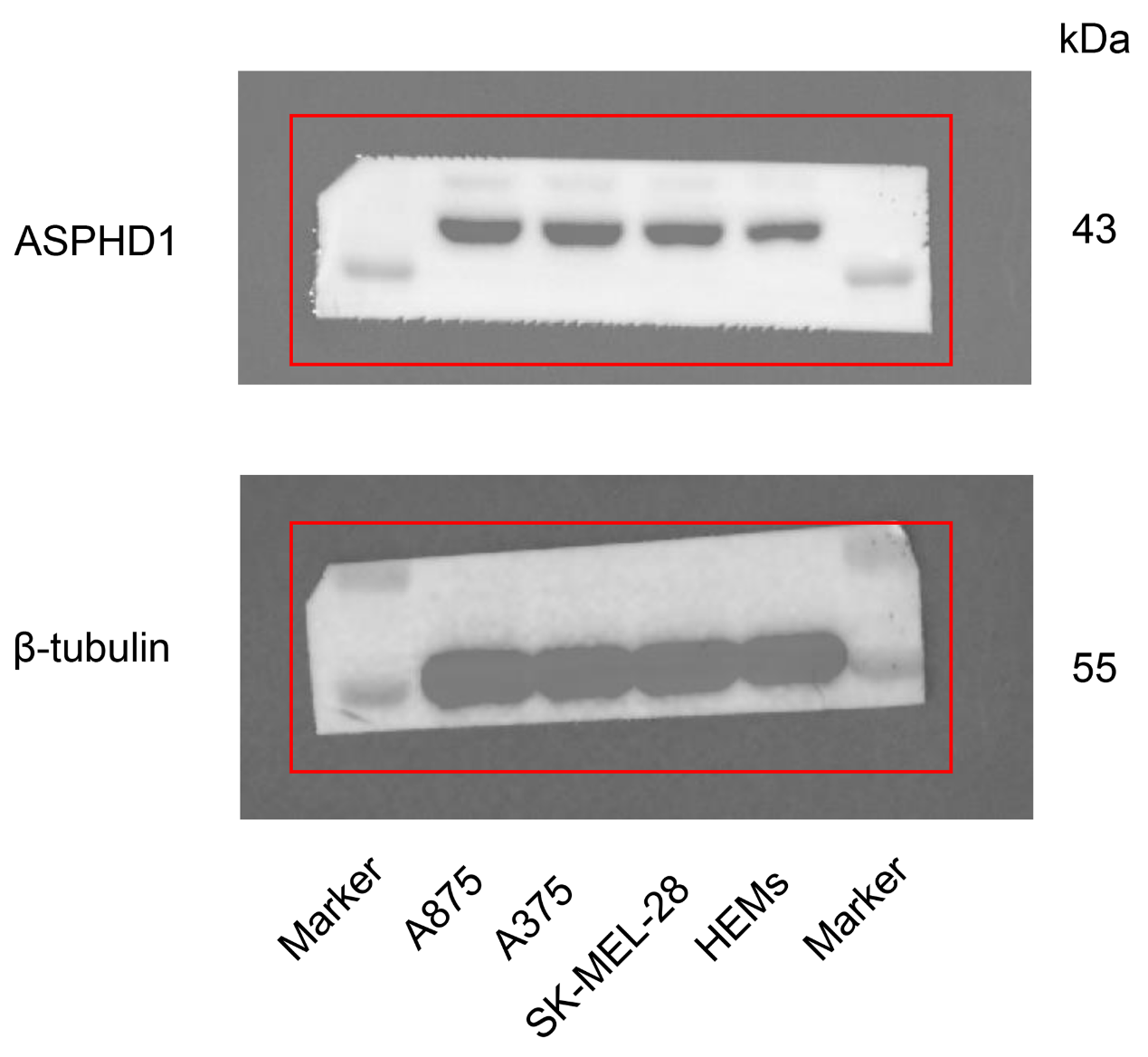


**Figure S4.** The original blots of ASPHD1 and β-tubulin for the third replicated experiment. Because the blots were cut before hybridisation with antibodies, the original images of full-length blots were absent.
